# Gangliocytic paraganglioma of the spine

**DOI:** 10.4322/acr.2021.277

**Published:** 2021-05-06

**Authors:** Shubha Lal, Ishita Pant, Sujata Chaturvedi, Pragyan Sarma

**Affiliations:** 1 Institute of Human Behaviour and Allied Sciences, Department of Pathology, New Delhi, India; 2 Guru Teg Bahadur Hospital, Department of Neurosurgery, New Delhi, India

**Keywords:** gangliocytic paraganglioma, spine

## Abstract

Paragangliomas are rare, encapsulated, benign neuroendocrine tumors that can arise from the adrenal medulla or extra-adrenal paraganglia. Extra-adrenal paragangliomas may develop a gangliocytic component with ganglion cells (Gangliocytic paragangliomas). Nearly 25%of cauda equina paragangliomas are gangliocytic paragangliomas. Here, we describe the case of a 35-year-old male who presented with weakness of both lower limbs over the last two months. Radiological findings were suggestive of myxopapillary ependymoma. However, the histopathological examination revealed a tumor with cells arranged in sheets, papillae, lobules, and around vessels forming pseudo rosettes. Ganglion cells were seen in small groups and, also singly. Tumor cells were immunopositive for chromogranin, synaptophysin, and S-100. Ganglion cells were immunopositive for synaptophysin, NSE, and NFP. A final histological diagnosis of Gangliocytic paraganglioma (WHO grade I) was made. To date, only nine gangliocytic paraganglioma cases have been previously reported, and to the best of our knowledge, this is the largest gangliocytic paraganglioma.

## INTRODUCTION

Paragangliomas are rare, encapsulated, benign neuroendocrine tumors that can arise from the adrenal medulla or extra-adrenal paraganglia.^1^ Extra-adrenal paragangliomas have been reported in the head and neck region (glomus jugulare and carotid body tumors), ampulla of Vater, jejunum, gastric pylorus, and, rarely, cauda equina. Extra-adrenal paragangliomas may develop a gangliocytic component (gangliocytic paragangliomas), which consists of ganglion cell components.^2^ The duodenum is the most common site of gangliocytic paraganglioma.^3^ Nearly 25%of cauda equina paragangliomas are gangliocytic paragangliomas.^4^ Gangliocytic paraganglioma (GP) was first reported in 1957 as ganglioneuroma by Dahl et al.^2^ It was named gangliocytic paraganglioma by Kepes and Zacharias in 1971.^5^ Herein, we describe the case of a 35-year-old male with a large gangliocytic paraganglioma of the spine. To date, only nine cases of gangliocytic paraganglioma have been previously reported, and to the best of our knowledge, this is the largest gangliocytic paraganglioma that has been reported.^1^^-^^6^

## CASE REPORT

**Figure 1:**
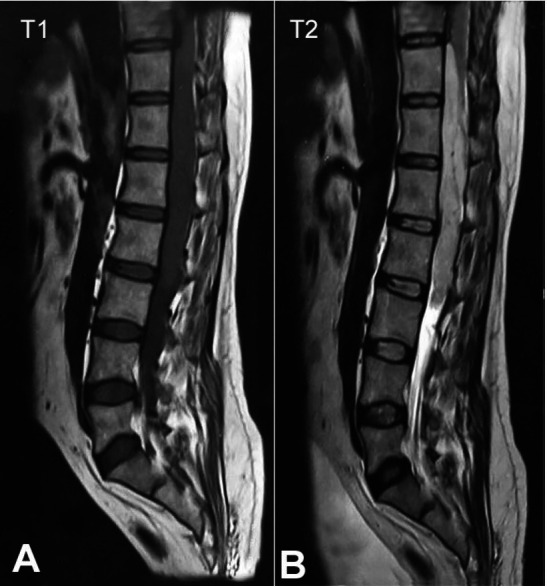
Spinal MRI. **A –** T1 weighted image – isointense intradural mass measuring 120 × 16 × 25 mm in conus medullaris and filum terminale from D11-L2 without local invasion into surrounding structures; **B –** T2 weighted image.

A 35-year-old male presented with lower limb weakness over the last two months. The spinal MRI showed a large enhancing intradural lesion, isointense on T1 weighted images, and hyperintense on T2 weighted images with homogeneous contrast enhancement extending from D11 to L2. The lesion measured 120 × 16 × 25 mm, encased the filum terminale, and compressed the conus region posteriorly. Mild scalloping of D11-L1 vertebral bodies was seen. A mild bulge into the neural foramina at D11-L1 and L1-2 was seen (Figures 1A and 1B). Radiological findings were suggestive of ependymoma.

**Figure 2:**
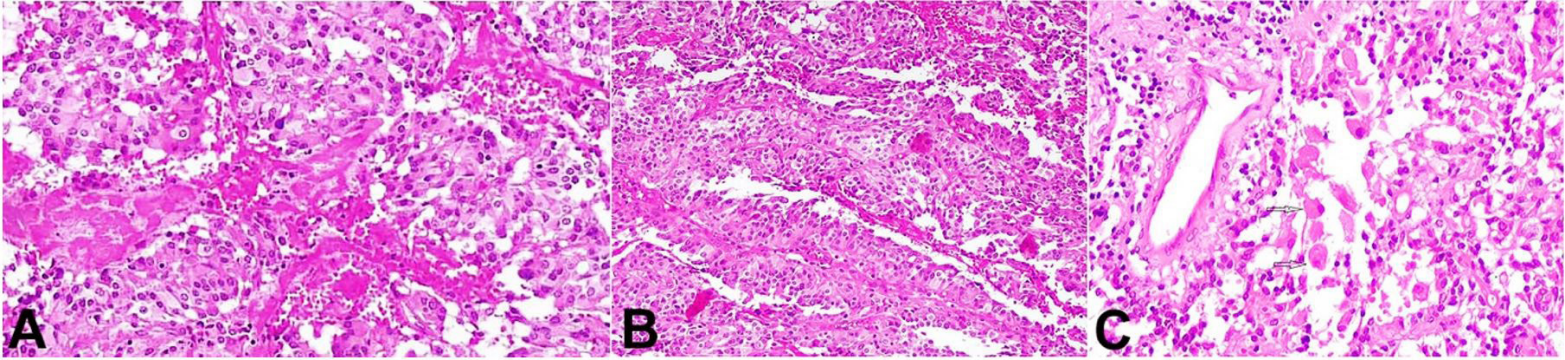
Photomicrographs of the tumor. Gangliocytic paraganglioma **A –** Tumor cells arranged in lobules (H&E, 200X); **B –** Ganglion arranged in papillae (H&E, 200X); **C –** Ganglion cells seen singly scattered (H&E, 200X).

**Figure 3:**
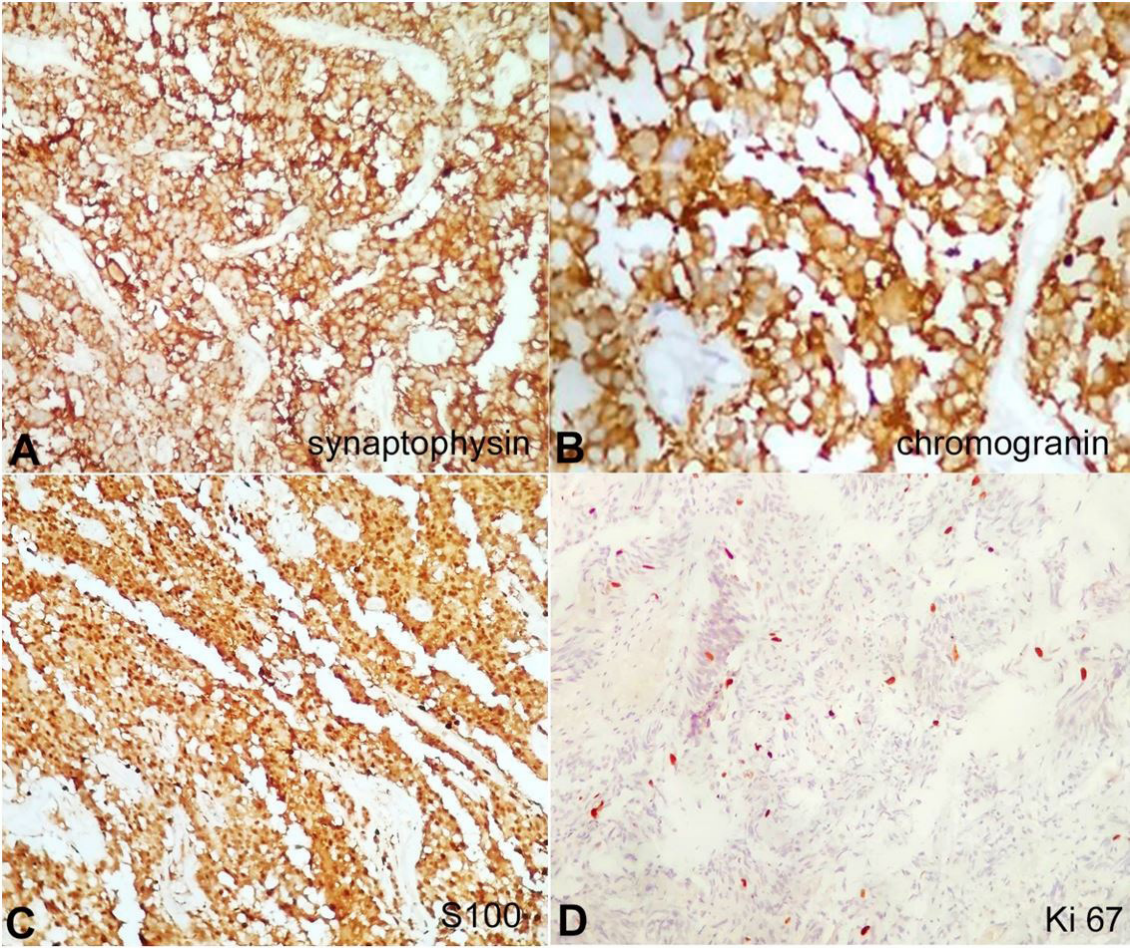
Photomicrographs of the tumor. Immunohistochemical panel. **A –** Tumor cells positive for synaptophysin (200X); **B –** Tumor cells positive for chromogranin (200X); **C –** Tumor cells positive for S100 (100X); **D –** Ki 67 proliferation index 8 – 10% in areas of highest proliferation (100X).

Complete surgical resection of the tumor was done. The histopathological examination revealed a tumor with cells arranged in sheets, papillae, lobules, and around vessels forming pseudo rosettes (Figures 2A and 2B). Various cell types were identified. In some areas, epithelial looking cohesive cells were seen in a lobular arrangement, highlighted on the reticular stain. Small bundles of spindly cells were seen interspersed in a fascicular arrangement. Ganglion cells were seen in small groups and, also singly (Figure 2C). The vasculature was prominent. The tumor cells arranged in lobules and papillae were immunopositive for chromogranin and synaptophysin (Figures 3A and 3B), while the spindle-shaped cells were immunopositive for S-100 (Figure 3C). Ganglion cells were immunopositive for synaptophysin, NSE, and NFP. The cells were immunonegative for GFAP, and Nestin. Ki-67 proliferation index was 8-10% in areas showing the highest proliferation. A final histological diagnosis of Gangliocytic Paraganglioma (WHO grade I) was made.

## DISCUSSION

According to the World Health Organization (WHO) classification, paragangliomas are grade I tumors developed from neural crest cells. By extension, paragangliomas located outside the adrenal gland have been designated as extra-adrenal paragangliomas. The spine is a rare site for these tumors.^6^ Paragangliomas in the spine are less infiltrative than their counterparts in the head and neck region. Nearly 25% of the spinal paragangliomas are seen to have a gangliocytic component. The origin of the gangliocytic variant remains unclear. It is believed to originate from neuroectodermal ganglion or spindle cells.^1^^,^^5^ The age of the patients with spinal paragangliomas ranges from 9 to 74 years, with most cases being diagnosed in middle age, a slight predominance has been noted in males, male/female = 1.4/1^7^^,^^8^ (Table 1).

**Table 1:** Literature review of gangliocytic paraganglioma of spine

Author	Age/sex	Clinical presentation	Site	Size (mm)	Histopathological findings	Surgical treatment
Present case	35/M	Weakness both legs	D11-L2	120 × 16 × 25	Neuroendocrine cells in Zellballen pattern, ganglion cells, S100 (+), synaptophysin (+), NSE(+),chromgranin (+),NFP (+)	Complete surgical resection
Nagose et al.^3^	42/M	difficulty in walking, pain and tingling sensation in the right leg	D12-L2	-	Neuroendocrine cells in Zellballen pattern, ganglion cells,schwann cells, S100 (+), synaptophysin (+), NSE(+),chromgranin (+)	Complete surgical resection
Akbik et al.^1^	68/M	Temporary urinary incontinence, LE, perineal paresthesias	S1-S2 intradural	60 × 26	Neuroendocrine cells in Zellballen pattern, ganglion cells, abundant cytoplasm, GFAP (−), S100 (+), synaptophysin (+)	Complete surgical resection
Vural et al.^2^	17/M	Low back pain, sciatica, difficulty in ambulation	L4 intradural	50 × 30	Neuroendocrine cells in Zellballen pattern, ganglion cells, calcification, GFAP (−), S100 (+)	Complete surgical resection
Llena et al.^4^	42/M	Low back pain, LE weakness	L1 intradural	35 × 20	Neuroendocrine cells in Zellballen pattern, large neurons, neurosecretory granules, dopamine (+)	Complete surgical resection
Matschke et al.^9^	63/F	Low back pain Cauda equina			Neuroendocrine cells in Zellballen pattern, vascular tissue, ganglion cells, GFAP (+)	Complete surgical resection
Djindjian et al.^10^	36/M	Low back pain, sudden paraplegia following sacral infiltration of medication	L2-L5 intradural	80 × 30	Cells in Zellballen pattern, large mature neurons, gangliocytic differentiation, neurosecretory granules	Complete surgical resection
Mishra et al.^5^	2 cases	Details not available	_	_	_	_
Moran et al.^6^	1 case	Details not available	_	_	_	_

The clinical features are usually characterized by lumbar pain, sciatica from mass effect, motor or sensory loss in lower extremities, or bowel and bladder dysfunction. The spinal paragangliomas are highly vascularized. The majority (75%) of paragangliomas are encapsulated, usually attached to the filum terminale or, less commonly, a nerve root.^3^ These tumors are intradural extramedullary in location. On MRI, the spinal paragangliomas are typically hypo- to isointense on T1-weighted images, hyperintense on T2-weighted images, and vividly enhancing on contrast studies. Other tumors of the cauda equina, including meningioma, schwannoma, and myxopapillary ependymoma, may show similar imaging profiles making the histologic examination the key to diagnosis. In some lesions, the characteristic “salt and pepper” appearance on T2 weighted images has been described due to the flow voids interspersed in a matrix of increased signal intensity caused by slow flow and tumor cells.^1^^,^^11^

All paragangliomas consist of two types of cells; type I and type II. The main components are lobules or nests of the chief cells (type I); known as Zellballen. They are surrounded by a single layer of sustentacular cells (type II). The histomorphological features of gangliocytic paraganglioma are similar to paraganglioma and ganglioneuroma.^12^ Immunohistochemical reactions for synaptophysin and neuron-specific enolase highlight the chief cells, while the sustentacular cells show expression of S100. The ganglion cells express synaptophysin, neuron- specific enolase, and neurofilament, indicating their neuronal differentiation. Glial fibrillary acidic protein can differentiate these tumors from the ependymoma, which is the most common differential diagnosis. Ependymal cells are GFAP positive, while neoplastic cells of paraganglioma are GFAP negative.^1^

The usual management of spinal paragangliomas is surgical resection. When the lesions are placed in the junction areas, laminotomy is preferred over laminectomy, considering the motion segments and potential for future instability. The role of adjuvant radiotherapy is controversial and should be reserved for unencapsulated or incompletely excised lesions, as it does not guarantee the prevention of tumor recurrence. However, follow-up in the form of a clinical examination every month after the operative procedure till six months, followed by every six months, and a radiological examination (MRI at third postoperative month followed by every six months) is recommended.^3^ The postoperative survival rate is good in spinal paragangliomas with a low recurrence rate. In their review, Gelabert-González^13^ reported a local recurrence rate of 2.2% in total excision cases and 5.4% to 10.5% in cases of subtotal excision of spinal paragangliomas. In the study by Yin et al.^14^ comprising 18 patients with spinal paragangliomas, they observed recurrence in only one patient. Mishra et al.^5^ reported no recurrence in eight patients with spinal paragangliomas.

## CONCLUSION

Spinal paraganglioma is seldom considered in preoperative differential diagnosis due to its rarity and nonspecific clinical features. Furthermore, because of the lack of pathognomonic radiologic features, they are frequently misdiagnosed as schwannoma or ependymoma. Complete surgical resection is considered curative and subtotal resection often leads to recurrence. Further, paragangliomas of this region may exhibit prominent areas of ependymoma-like histology. Hence although rare, paragangliomas should be included in the differential diagnosis of an intradural, extramedullary tumor of this region, and IHC/ultrastructural studies should be done for accurate diagnosis in doubtful cases.

We have described a case of a 35-year-old male with a large gangliocytic paraganglioma of the spine, diagnosed as ependymoma radiologically. To date, only nine cases of gangliocytic paraganglioma have been previously reported, and this is the largest gangliocytic paraganglioma to have been reported.
